# Patterns of Alcohol Consumption Among Individuals With Alcohol Use Disorder During the COVID-19 Pandemic and Lockdowns in Germany

**DOI:** 10.1001/jamanetworkopen.2022.24641

**Published:** 2022-08-01

**Authors:** Friederike Deeken, Markus Reichert, Hilmar Zech, Julia Wenzel, Friederike Wedemeyer, Alvaro Aguilera, Acelya Aslan, Patrick Bach, Nadja S. Bahr, Claudia Ebrahimi, Pascale C. Fischbach, Marvin Ganz, Maria Garbusow, Charlotte M. Großkopf, Marie Heigert, Angela Hentschel, Damian Karl, Patricia Pelz, Mathieu Pinger, Carlotta Riemerschmid, Annika Rosenthal, Johannes Steffen, Jens Strehle, Franziska Weiss, Gesine Wieder, Alfred Wieland, Judith Zaiser, Sina Zimmermann, Henrik Walter, Bernd Lenz, Lorenz Deserno, Michael N. Smolka, Shuyan Liu, Ulrich W. Ebner-Priemer, Andreas Heinz, Michael A. Rapp, and the ReCoDe Consortium

**Affiliations:** 1 Department of Social and Preventive Medicine, University of Potsdam, Potsdam, Germany; 2 Department of Psychiatry and Psychotherapy, Central Institute of Mental Health, Heidelberg University, Medical Faculty Mannheim, Mannheim, Germany; 3 Mental mHealth Lab, Department of Sports and Sports Science, Karlsruhe Institute of Technology, Karlsruhe, Germany; 4 Department of eHealth and Sports Analytics, Faculty of Sports Science, Ruhr-University Bochum, Bochum, Germany; 5 Department of Psychiatry, Technische Universität Dresden, Dresden, Germany; 6 Department of Psychiatry and Neurosciences, Charité–Universitätsmedizin Berlin, corporate member of FreieUniversität Berlin and Humboldt-Universität zu Berlin, Berlin, Germany; 7 Epilepsy-Center Berlin-Brandenburg, Epilepsy-Clinic Tabor, Bernau, Germany; 8 Center for Information Services and High Performance Computing, Technische Universität Dresden, Dresden, Germany; 9 Department of Addictive Behavior and Addiction Medicine, Central Institute of Mental Health, Medical Faculty Mannheim, Heidelberg University, Mannheim, Germany; 10 Department of Clinical Psychology, Central Institute of Mental Health, Medical Faculty Mannheim, Heidelberg University, Mannheim, Germany; 11 Department of Child and Adolescent Psychiatry, Psychotherapy and Psychosomatics, University of Würzburg, Würzburg, Germany; 12 Max Planck Institute for Human Cognitive and Brain Sciences, Leipzig, Germany

## Abstract

**Importance:**

Alcohol consumption (AC) leads to death and disability worldwide. Ongoing discussions on potential negative effects of the COVID-19 pandemic on AC need to be informed by real-world evidence.

**Objective:**

To examine whether lockdown measures are associated with AC and consumption-related temporal and psychological within-person mechanisms.

**Design, Setting, and Participants:**

This quantitative, intensive, longitudinal cohort study recruited 1743 participants from 3 sites from February 20, 2020, to February 28, 2021. Data were provided before and within the second lockdown of the COVID-19 pandemic in Germany: before lockdown (October 2 to November 1, 2020); light lockdown (November 2 to December 15, 2020); and hard lockdown (December 16, 2020, to February 28, 2021).

**Main Outcomes and Measures:**

Daily ratings of AC (main outcome) captured during 3 lockdown phases (main variable) and temporal (weekends and holidays) and psychological (social isolation and drinking intention) correlates.

**Results:**

Of the 1743 screened participants, 189 (119 [63.0%] male; median [IQR] age, 37 [27.5-52.0] years) with at least 2 alcohol use disorder (AUD) criteria according to the *Diagnostic and Statistical Manual of Mental Disorders* (Fifth Edition) yet without the need for medically supervised alcohol withdrawal were included. These individuals provided 14 694 smartphone ratings from October 2020 through February 2021. Multilevel modeling revealed significantly higher AC (grams of alcohol per day) on weekend days vs weekdays (β = 11.39; 95% CI, 10.00-12.77; *P* < .001). Alcohol consumption was above the overall average on Christmas (β = 26.82; 95% CI, 21.87-31.77; *P* < .001) and New Year’s Eve (β = 66.88; 95% CI, 59.22-74.54; *P* < .001). During the hard lockdown, perceived social isolation was significantly higher (β = 0.12; 95% CI, 0.06-0.15; *P* < .001), but AC was significantly lower (β = −5.45; 95% CI, −8.00 to −2.90; *P* = .001). Independent of lockdown, intention to drink less alcohol was associated with lower AC (β = −11.10; 95% CI, −13.63 to −8.58; *P* < .001). Notably, differences in AC between weekend and weekdays decreased both during the hard lockdown (β = −6.14; 95% CI, −9.96 to −2.31; *P* = .002) and in participants with severe AUD (β = −6.26; 95% CI, −10.18 to −2.34; *P* = .002).

**Conclusions and Relevance:**

This 5-month cohort study found no immediate negative associations of lockdown measures with overall AC. Rather, weekend-weekday and holiday AC patterns exceeded lockdown effects. Differences in AC between weekend days and weekdays evinced that weekend drinking cycles decreased as a function of AUD severity and lockdown measures, indicating a potential mechanism of losing and regaining control. This finding suggests that temporal patterns and drinking intention constitute promising targets for prevention and intervention, even in high-risk individuals.

## Key Points

**Question:**

Are COVID-19 lockdown measures associated with alcohol consumption (AC) and temporal patterns of AC?

**Findings:**

In this cohort study of 189 participants who met the criteria for alcohol use disorder (AUD), high-frequency AC tracking comprising 14 694 smartphone ratings revealed no immediate negative association of lockdown measures with overall AC. Independent of the lockdown, intention to control AC was associated with less AC; however, a difference between AC on weekends vs weekdays decreased during lockdown measures and in individuals with severe AUD.

**Meaning:**

Both holidays and weekly patterns were associated with drinking intention and lockdown measures, reflecting losing and regaining control over AC; these patterns may serve as targets for prevention and intervention of AUD.

## Teaser

This cohort study of individuals with alcohol use disorder examines whether COVID-19 lockdown measures in Germany were associated with changes in alcohol consumption.

## Introduction

Alcohol use is one of the leading causes of mortality and morbidity worldwide.^1,2,3^ Potential negative effects of the ongoing COVID-19 pandemic on alcohol consumption (AC) have been previously discussed.^4,5,6^ Social distancing measures, exit restrictions, and altered work and family conditions that were mandatory to mitigate the pandemic may trigger increases in AC, especially in people at risk for substance use disorders.^7^ To date, most studies examining the association of the pandemic with drinking behavior have relied on retrospective assessments,^8,9^ although methodologic shortcomings of retrospective AC assessments and psychological variables are well known.^10,11,12^ For example, retrospective methods cannot reliably capture alcohol use, yet real-time assessments of AC can capture more drinking days, a higher AC intake, a greater number of binge episodes, and a faster AC consumption rate compared with retrospective AC reporting.^12^ However, real-world evidence on AC during the pandemic remains elusive, and studying health behaviors in real life is critically warranted.^13^ Therefore, to quantify the potential influences of pandemic measures, we used smartphone-based daily ratings of AC and its temporal (weekends and holidays) and psychological (social isolation and drinking intention) everyday life correlates captured across a 5-month period in participants who met criteria for alcohol use disorder (AUD).

## Methods

### Study Procedure

For this quantitative, intensive, longitudinal cohort study, participants were recruited online and through advertisements and flyers at 3 sites in Germany (Charité–Universitätsmedizin Berlin, Technical University Dresden, and Central Institute of Mental Health in Mannheim) (eAppendix 1 in Supplement 1).^14^ A telephone screening was performed to verify inclusion and exclusion criteria. Eligible participants were scheduled for a basic assessment appointment during which baseline data were collected and a customized ecological momentary assessment (EMA) smartphone application (movisens app, movisens GmbH) was installed.^15^ All participants gave written informed consent before participating in the study. For participants younger than 18 years, a parent or guardian had to provide cosignature of consent. The study was approved by the review boards of the local ethics committees at Heidelberg University, Charité–Universitätsmedizin Berlin, and Technical University Dresden. Participants were compensated monetarily for their participation. This study followed the Strengthening the Reporting of Observational Studies in Epidemiology (STROBE) reporting guideline.

### Inclusion and Exclusion Criteria

A total of 1743 individuals were screened. Individuals were included if they were between 16 and 65 years of age with the presence of at least 2 criteria for AUD according to the *Diagnostic and Statistical Manual of Mental Disorders* (Fifth Edition) (*DSM-5*)^16^ yet without the need for medically supervised alcohol withdrawal and no desire for therapeutic intervention, had sufficient German language skills, were able to understand the study protocol and give informed consent, and were willing to use an Android smartphone. Exclusion criteria were current use of drugs or medications that interact with the central nervous system; contraindications to magnetic resonance imaging; medical history of *DSM-5* bipolar disorder, psychotic disorder, schizophrenia or schizophrenic spectrum disorder, or substance dependence other than alcohol, nicotine, or cannabis; and medical history of severe head injury or other severe central nervous system disorders.

### Data Assessment

All participants completed a battery of interviews and self-report questionnaires. Criteria for AUD were assessed using the clinical version of the Structured Clinical Interview for the *DSM-5*.^16^ Sociodemographic data (eg, sex, age, and years of education) were collected. A total of 189 participants (65 from Berlin, 28 from Dresden, and 96 from Mannheim) who were recruited between February 20, 2020, and February 28, 2021, and had valid information in baseline data and EMA data were eligible for the current study (eAppendix 2 in Supplement 1). To avoid detection bias, we ensured that all assessors were well trained.

### EMA and Electronic Diary Items

To assess real-time AC (primary outcome) and its potential everyday life correlates, we asked participants to install a movisensXS app that included electronic diaries (e-diary) (eAppendix 3 in Supplement 1). We provided participants with smartphones (Nokia 3.4, Nokia 6.2, and Nokia 7.2) for research purposes in case their own smartphones were incompatible. Participants were prompted to complete an e-diary entry via the app once every second day. An acoustic, vibration, and visual alarm was triggered at 12 pm but could be postponed up to 8 hours until 8 pm (in 5-minute to 8-hour intervals). If the alarm was ignored, it recurred 5 more times in 90-minute intervals, and a button to start the e-diary remained on the home screen of the app throughout the day to enable participants to start the questionnaire individually.

For the current study, approximately every other day at 12 pm, participants rated AC, perceived social isolation, and their intention to drink on smartphone-based e-diaries with good compliance rates (AC data for a median of 5.83 [IQR, 3.74-6.77] days per week were provided) (Table 1) as they went about their daily routines. The items were displayed in the German language.

**Table 1.** Participant Characteristics and EMA Descriptives

Characteristic	Finding^a^ (N = 189)
Age, median (IQR) [range], y	37 (27.5-52.0) [17-65]
Sex	
Male	119 (63.0)
Female	70 (37.0)
Total No. of AUD criteria, median (IQR) [range]	4 (3-5) [2-7]
No. of AUD criteria	
2	40 (21.2)
3	50 (26.5)
4	33 (17.5)
5	41 (21.7)
6	18 (9.5)
7	7 (3.7)
Highest school qualification	
Pupil at a general education school	7 (3.7)
Currently enrolled in career-based training	1 (0.5)
Secondary general school certificate (Hauptschulsabschluss)	4 (2.1)
General Certificate of Secondary Education (Realschulsabschluss)	26 (13.7)
Polytechnic secondary school (Abschluss polytechnische Oberschule)	2 (1.1)
Advanced technical college certificate (Fachhochschulreife)	22 (11.6)
General Certificate of Education (Abitur)	116 (61.3)
Another school degree	3 (1.6)
Marital status	
Single	87 (46.0)
Living in marriage or partnership	75 (39.6)
Living separately	10 (5.3)
Divorced	7 (3.7)
Widowed	2 (1.1)
EMA compliance, median (IQR)^b^	83.33 (53.44-96.67)
EMA perceived social isolation, median (IQR)^c^	1.13 (1.00-1.71)
EMA drinking intention^d^	
No particular resolutions	6671 (31.8)
No more than usual	3593 (17.2)
Less than usual	4040 (19.3)
EMA alcohol consumption, median (IQR), g/d	31.06 (18.37-44.56)

Abbreviations: AUD, alcohol use disorder; EMA, ecological momentary assessment.

^a^ Data are presented as number (percentage) of patients unless otherwise indicated.

^b^ The compliance rate is calculated based on the data points of alcohol consumption ratings per day and equals a median of 5.83 (IQR, 3.74-6.77) days per week for which participants provided their alcohol consumption data.

^c^ Four-point Likert scale from 1 (does not apply) to 4 (fully applies).

^d^ Frequencies of EMA intention ratings.

**Figure 1.**
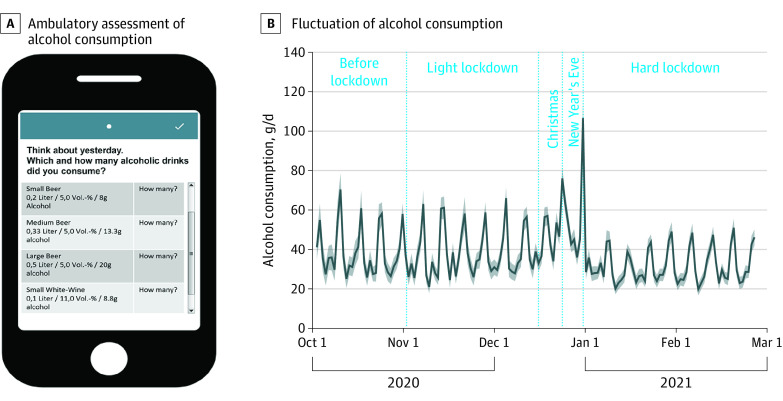
Assessment of Alcohol Consumption and Its Fluctuation During the Second Wave of the COVID-19 Pandemic in Germany Lockdown measures (beginning of the light lockdown and beginning of the hard lockdown) as well as holidays (Christmas and New Year’s Eve) are highlighted. Solid line indicates the mean; shaded area, SD.

Alcohol intake (ie, our primary outcome) during the past 2 days was assessed for each day separately (Figure 1A): “Think about yesterday (second item: the day before yesterday): Which and how many alcoholic drinks did you consume? (Denken Sie an gestern [second item: Denken Sie an vorgestern]: Welche und wie viele alkoholische Getränke haben Sie konsumiert?).” Participants were asked to select from a list of alcoholic drinks with an indication of the amount of alcohol by volume plus an input field to indicate the number of respective drinks consumed (eTable 1 in Supplement 1). Compared with retrospective AC reporting, a prominent cross-validation study^12^ showed that e-diary ratings capture more drinking days, a higher AC intake, a greater number of binge episodes, and a faster AC consumption rate, thereby increasing the AC report reliability.

Feelings of social isolation (secondary outcome) were assessed on a 4-point Likert scale from 1, indicating does not apply (Trifft nicht zu), to 4, indicating fully applies (Trifft völlig zu), with the question, “During the past 24 hours I felt isolated from others (In den letzten 24 Stunden fühlte ich mich von anderen isoliert),” an established item derived from Fydrich et al^17^ (eFigure 1A in Supplement 1).

Intention to limit drinking (within-person variable) was assessed with the question, “Do you plan to limit your alcohol consumption for the next 8 days? (Planen Sie, Ihren Alkoholkonsum die nächsten 8 Tage zu begrenzen?)”, with 1 indicating “No, I don’t have any particular resolutions (Nein, ich habe keine speziellen Vorsätze)”, 2 indicating “Yes, I want to drink not more than usual (Ja, ich möchte nicht mehr als üblich trinken)”, and 3 indicating “Yes, I want to drink less than usual (Ja, ich möchte weniger als üblich trinken)” (eFigure 1B in Supplement 1).

### COVID-19 Lockdown Definitions and Governmental Measures

Data were provided before and within the second lockdown of the COVID-19 pandemic in Germany: before lockdown (October 2 to November 1, 2020); light lockdown (November 2 to December 15, 2020), a period with restrictions such as a maximum of 10 people being allowed to meet; and hard lockdown (December 16, 2020, to February 28, 2021), when a maximum of 5 people were allowed to meet and AC in public areas was prohibited (eTable 2 in Supplement 1).

To contain the COVID-19 pandemic, governmental measures were implemented in Germany.^18,19^ Because of the increased spread of the virus in the fall, a light lockdown included contact restrictions to a maximum of 10 people as well as the closing of bars and restaurants. Because of a further increase in infections, further restrictions were implemented (eg, restriction of meetings to a maximum of 5 people and a prohibition on the consumption of alcoholic beverages in public areas) (eTable 2 in Supplement 1).

### Statistical Analysis

To investigate potential correlates of AC (primary outcome), we subjected the data to multilevel models by nesting e-diary ratings (level 1) within participants (level 2),^20^ setting the α level to .05, testing 2-sided hypotheses, and using SAS statistical software, version 9.4 (SAS Institute Inc) (eAppendixes 4-6 in Supplement 1). Multilevel modeling is robust against missing data.^20^ For our main model, we entered the dichotomous level 1 variables of weekend (Friday to Sunday vs Monday to Thursday), Christmas (December 24-26 vs all other days), and New Year’s Eve (December 31 vs all other days), the categorical level 1 variables of lockdown measures (before vs light vs hard) and drinking intention (no particular resolutions vs no more than usual AC vs less than usual AC), as well as the dimensional level 1 variable of perceived social isolation into the model. Following established procedures,^20^ we centered the variable of perceived social isolation on the persons’ mean. The dimensional level 2 variables of age, sex, AUD criteria, and the categorical variable of study center (Mannheim, Dresden, or Berlin) were incorporated as covariates to the multilevel statistic:

*Y *(Alcohol Consumption)*_ij_* = β*_00_* + β*_01_* × Age*_j_* + β*_02_* × Sex*_j_* + β*_03_* × AUD Criteria*_j_* + β*_04_* × Study Center*_j_* + β*_10_* × Weekend*_ij_* + β*_20_* × Christmas*_ij_* + β*_30_* × New Year’s Eve*_i_*_j_ + β*_40_* × Lockdown Measures_ij_ + β*_50_* × Perceived Social Isolation*_ij_* + β*_60_* × Drinking Intention*_ij_* + *μ_ij_* + *r_ij_*

Here, within-person effects on level 1 were estimated from participants’ e-diary entries (subscript *j*) across the measurement time points (subscript *i*). The level of AC in participant *j* at time *i* is indicated as *Y_ij_*. The intercept as well as the effects of the weekend, Christmas, New Year’s Eve, lockdown measures, perceived social isolation, and drinking intention at level 1 are depicted by the β coefficients; *r_ij_* represents the level 1 residuals. Between-person effects were estimated on level 2, and β coefficients depict the effects of age, sex, AUD criteria, and study center on participants’ AC. The individual variation of effects around the fixed effect (ie, random effects) are depicted by *μ_ij_*. Because ignoring potential autocorrelations violates statistical assumptions (SEs of fixed effects are too low and test statistics are too large, which may lead to a type I error), we included autoregressive error terms.

## Results

Of the 1743 screened participants, 189 individuals (119 [63.0%] male and 70 [37.0%] female; median [IQR] age, 37 [27.5-52.0] years) with at least 2 AUD criteria according to the *DSM-5* yet without the need for medically supervised alcohol withdrawal were included. Participants had 14 694 valid e-diary entries from October 2020 through February 2021 (eFigure 2 in Supplement 1). Detailed participant characteristics are given in Table 1 and eTable 3 in Supplement 1.

**Figure 2.**
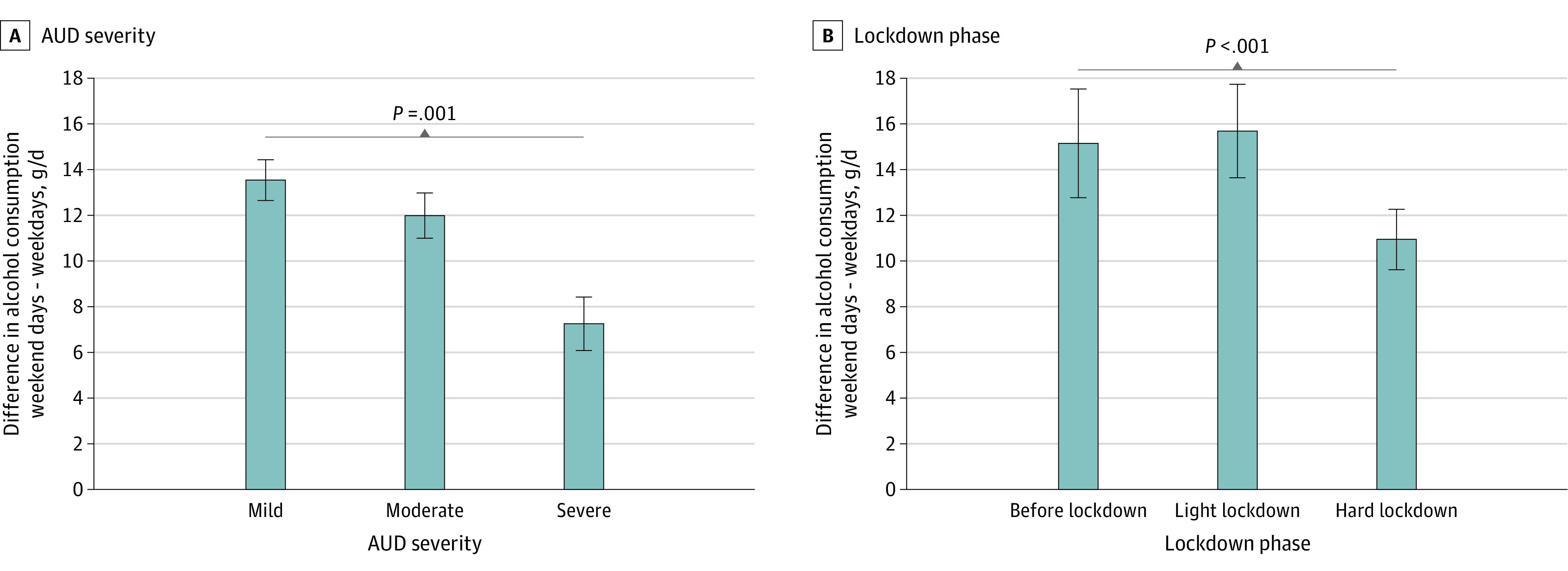
Differences in Weekend Drinking Cycles Moderated by Alcohol Use Disorder (AUD) Severity and Lockdown Phase

**Figure 3.**
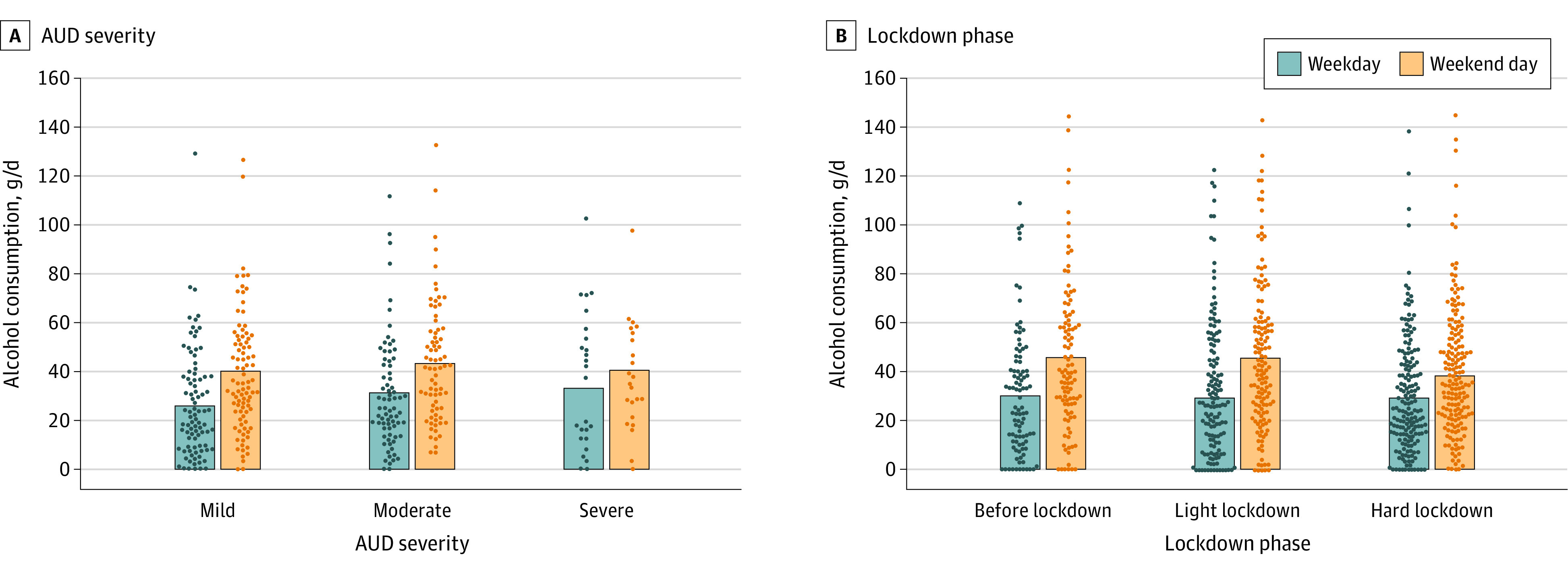
Alcohol Consumption on Weekdays vs Weekend Days by Alcohol Use Disorder (AUD) Category and Lockdown Phase Dots indicate averaged raw data (ie, mean alcohol consumption per participant on weekday vs weekend day); bars indicate the group mean.

Across the study period of 5 months, participants consumed a median of 31.06 g (IQR, 18.37-44.56 g) of alcohol per day (ie, approximately 3 standard drinks,^21^ such as 400 mL of wine). Overall, we found a distinct weekly drinking pattern in that on weekend days, median AC was 11.39 g (95% CI, 10.00-12.77 g; *P* < .001) higher per day compared with weekdays (Figure 1) (ie, approximately 1 standard drink, such as 1 small glass of regular beer). Daily AC was above the overall median on Christmas by 26.82 g (95% CI, 21.87-31.77 g; *P* < .001) (approximately 2 standard drinks) and on New Year’s Eve by 66.88 g (95% CI, 59.22-74-54 g; *P* < .001) (approximately 5 standard drinks) (Table 2; eTables 4-6 and eFigure 4 in Supplement 1). In line with increased social distancing measures, perceived social isolation was significantly heightened during the hard lockdown vs before lockdown (β = 0.12; 95% CI, 0.06-0.15; *P* < .001) (eFigure 3 in Supplement 1), whereas it was not altered during the light lockdown vs before lockdown (eTable 7 in Supplement 1). However, perceived social isolation was not associated with AC (β = −1.31; 95% CI, −2.89 to 0.27; *P* = .10) (Table 2). During the hard lockdown, AC was −5.45 g/d (95% CI, −8.00 to −2.90 g/d; *P* = .001) lower compared with before lockdown (ie, approximately half a standard drink) (Table 2). In contrast, AC was unchanged during the light lockdown vs before lockdown (Table 1). A higher intention to drink less alcohol was associated with significantly lower AC (β = −11.10; 95% CI, −13.63 to −8.58; *P* < .001) (Table 2) regardless of the lockdown phase (*F*_3967_ = 0.63; *P* = .64) (eTable 8 in Supplement 1). In particular, if participants intended to drink less than usual for the upcoming 8 days compared with not having any particular resolutions, their self-reported AC was decreased by 11.10 g (95% CI, −13.63 to −8.58 g) per day within this time frame. The difference of AC between weekend days and weekdays was associated with AUD severity (*F*[12 × 10^3^] = 4.89; *P* = .008), that is, participants with more severe AUD showed a significantly lower difference of AC between weekend days and weekdays (Figure 2A; eTables 9 and 10 in Supplement 1). In particular, AC on weekdays was higher in those with severe AUD compared with those with mild AUD (β = −6.26; 95% CI, −10.18 to −2.34; *P* = .002), yet consumption on weekend days was similar in both groups (Figure 3A). The weekend-weekday difference in AC was also associated with the lockdown phases (*F*[12 × 10^3^] = 11.96; *P* < .001), that is, it was significantly lower during the hard lockdown phase compared with before lockdown (β = −6.14; 95% CI, −9.96 to −2.31; *P* = .002) (Figure 2B; eTable 11 in Supplement 1). In particular, AC on weekend days was lower during the hard lockdown compared with before lockdown and during the light lockdown, but AC on weekdays was similar across all lockdown phases (Figure 3B).

**Table 2.** Key Multilevel Modeling Results

Variable	Outcome: alcohol consumption		
	β (SE) [95% CI]	*t* (*df*)	*P* value
Intercept	8.95 (7.83) [−5.49 to 25.4]	1.27 (188)	.20
Age	0.48 (0.14) [0.20 to 0.75]	3.44 (183)	<.001
Sex			
Male	1 [Reference]	1 [Reference]	1 [Reference]
Female	−9.91 (3.52) [−16.86 to −2.97]	−2.82 (181)	.005
AUD criteria	3.41 (1.18) [1.09 to 5.74]	2.90 (179)	.004
Study center			
Central Institute of Mental Health in Mannheim	1 [Reference]	1 [Reference]	1 [Reference]
Charité–Universitätsmedizin Berlin	−3.26 (3.90) [−10.96 to 4.43]	−0.84 (178)	.40
Technical University Dresden	−0.16 (5.38) [−10.76 to 10.45]	−0.03 (183)	.98
Weekend vs weekday			
Weekday	1 [Reference]	1 [Reference]	1 [Reference]
Weekend	11.38 (0.71) [10.00 to 12.77]	16.09 (9015)	<.001
Christmas			
No Christmas (ie, all other days)	1 [Reference]	1 [Reference]	1 [Reference]
Christmas	26.82 (2.52) [21.87 to 31.77]	10.62 (6137)	<.001
New Year’s Eve			
No New Year’s Eve (ie, all other days)	1 [Reference]	1 [Reference]	1 [Reference]
New Year’s Eve	66.88 (3.91) [59.22 to 74.54]	17.11 (11 × 10^3^)	<.001
Lockdown stage			
Before lockdown	1 [Reference]	1 [Reference]	1 [Reference]
Light lockdown	−1.30 (1.34) [−3.94 to 1.33]	−0.97 (3539)	.33
Hard lockdown	−5.45 (1.30) [−8.00 to −2.90]	−4.19 (3523)	.001
Perceived social isolation	−1.31 (0.81) [−2.89 to 0.27]	−1.63 (6865)	.10
Intention			
No particular resolutions	1 [Reference]	1 [Reference]	1 [Reference]
No more AC than usual	−3.97 (1.32) [−6.56 to −1.38]	−3.00 (3940)	.003
Less AC than usual	−11.10 (1.29) [−13.63 to −8.58]	−8.63 (3979)	<.001

Abbreviations: AC, alcohol consumption; AUD, alcohol use disorder.

## Discussion

As an essential complement to the partially contradictory scientific and public debate about the effects of COVID-19 on health behaviors,^22,23^ this 5-month, study that includes innovative high-frequency tracking of AC in participants with mainly mild to moderate AUD provides real-world evidence that overall AC decreased within study participants during the hard lockdown of the COVID-19 pandemic in Germany. Independent of the pandemic measures, contextual and psychological correlates on AC were identified. Results showed a clear weekend drinking cycle with strongly increased AC on weekends and holidays as well as a close coupling of drinking intention and AC. These findings can inform prevention and therapeutic interventions in mild to moderate AUD, for example, by identifying drinking intention as a target for behavioral interventions even in high-risk individuals and as a potential behavioral mechanism that appeared independent of lockdown measures. Beyond these findings, via exploratory analyses, we found a flattening of the weekend drinking cycle as a function of both AUD severity and lockdown measures. This finding may indicate a pattern of losing and regaining control over alcohol intake, with individuals with severe AUD potentially differentiating less between working and nonworking days. At the same time, during the hard lockdown, when a maximum of 5 people were allowed to meet and AC in public areas was prohibited, lower AC at weekend days may indicate that participants consumed less alcohol as a result of contextual influences, which are particularly relevant to individuals with consumption behavior driven by social aspects. Social interaction and/or social isolation have been found to be associated with AC in different age cohorts.^24^ One may be tempted to speculate that contact restrictions could partially explain the reduced weekday-weekend AC difference in participants with severe AUD, who may drink alcohol not only in social contexts but also when alone. Therefore, weekend drinking cycles may serve as a promising change-sensitive marker of losing and regaining control over alcohol intake, a proposal that needs to be substantiated in further prevention and intervention studies.

### Limitations

This study has some limitations, and some aspects of this work merit further refinements in future research. First, because all data were captured during the COVID-19 pandemic, the generalizability of our results is limited. In addition, our sample is a nonrepresentative but selective convenience sample of mainly male individuals with mild to moderate AUD, which may further limit the generalizability of the findings. However, studies^25,26,27,28^ reveal that, without professional help, up to 19% of untreated patients with alcohol dependency manage to regain control over drug intake, becoming abstinent or substantially reducing their drug intake. In contrast, a small percentage of patients with severe alcohol dependence who require detoxification manage to control their AC on a low alcohol intake level over the long term.^29^ To this end, we focused on the broad and dimensional spectrum of patients with AUD without the need for inpatient detoxification. This sampling strategy enabled us to focus on aspects of losing and regaining control over alcohol intake. More closely, we aimed to examine a sample of people who are at some risk in terms of their AC but who are not yet manifestly dependent on alcohol. One may be tempted to speculate that this target population may be more prone to lockdown influences on drinking behavior compared with clinical patients or the general population.

Second, because we investigated the second COVID-19 phase in Germany, the prelockdown phase can also be seen as a postlockdown phase, which may confound the investigation of pandemic lockdown effects on drinking behavior. Although it is unclear whether this would lead to an overestimation vs underestimation of associations between lockdown measures and consumption, our data indicate that this did not impact the validity of the findings. Data show higher social isolation during the hard lockdown vs before lockdown, a plausible and expected effect of the lockdown (ie, people felt more socially isolated as a consequence of the lockdown). Moreover, robustness checks clearly showed that consumption rates in the postlockdown phase (May 9 to September 30, 2021) reached levels comparable with the prelockdown phase as confirmed by multilevel statistics, which supports the assumption that we indeed investigated lockdown effects on AC.

Third, there was a natural close coupling of the start of the hard lockdown, the Christmas season, and the turn of the year potentially bringing up good New Year’s resolutions, which might have affected AC. However, when statistically controlling for these associations with holidays and intentions, we still found significant decreases instead of the expected increases of AC during hard lockdown.

Fourth, although a series of supplementary analyses regarding potential between-person influences, lockdown parameterization, and the outcome distribution confirmed the robustness of the findings, our naturalistic observation precludes drawing causal conclusions because of potentially hidden third variables on specific triggers of AC changes^15^; therefore, experimental manipulation in everyday life is critically warranted to confirm positive influences of drinking intentions on AC.^30^

## Conclusions

In this cohort study of individuals with mild to moderate AUD, we found no immediate negative associations between lockdown measures and overall AC, indicating that lockdown measures alone may not aggravate consumption patterns in the observed population. Furthermore, we clearly delineated a pattern of weekend to weekday and holiday-related variation in drinking behavior as well as an association of AC with intention to drink, which is suggestive of habitual consumption patterns together with maintained control over intake in mild to moderate AUD. During hard lockdown measures on the one hand and in patients with more severe AUD on the other hand, weekday to weekend drinking cycles were altered, indicating a possible mechanism of losing and regaining control potentially modified by social contacts and AUD severity. In sum, our analyses on everyday life correlates of AC drawing from EMA suggest that temporal patterns and drinking intention constitute promising targets for prevention and intervention, especially in individuals at high risk for alcohol dependence.

## Notes

Supplement 1. **eAppendix 1. **Collaborative Research Center Grant 265 “ReCoDe” **eAppendix 2. **Participant Recruitment and Characteristics **eAppendix 3.** e-Diary Items **eAppendix 4. **Statistical Analyses and Results **eAppendix 5. **Main Multilevel Results **eAppendix 6. **Supportive Multilevel Analyses
eReferences
**eTable 1. **e-Diary List of Alcoholic Drinks **eTable 2. **Lockdown Measures in Germany **eTable 3. **Sex Distributed by Age and AUD Severity **eTable 4. **Main Multilevel Model Extended by Between-Person Predictors **eTable 5.** Holiday Season as an Additional Lockdown Phase Category **eTable 6. **Main Model With Log-Transformed Outcome **eTable 7. **Multilevel Results on Perceived Social Isolation **eTable 8. **Moderation Analyses Lockdown * Intention **eTable 9. **Moderation Analyses AUD Criteria * Weekend **eTable 10. **Moderation Analyses AUD Categories * Weekend **eTable 11. **Moderation Analyses Lockdown * Weekend **eFigure 1.** Repeated Real-life Assessment of (a) Perceived Social Isolation and (b) Drinking Intention **eFigure 2.** Study Flow Diagram **eFigure 3.** Development of Perceived Social Isolation (Y-Axis; Centered Within Subjects) During the Second Wave of the COVID-19 Pandemic in Germany **eFigure 4.** Level 1 Residuals

Supplement 2.
Nonauthor Collaborators

## References

[1] Griswold MG, Fullman N, Hawley C, ; GBD 2016 Alcohol Collaborators. Alcohol use and burden for 195 countries and territories, 1990-2016: a systematic analysis for the Global Burden of Disease Study 2016. Lancet. 2018;392(10152):1015-1035. doi:10.1016/S0140-6736(18)31310-2 30146330 PMC6148333

[2] Heinz A, Beck A, Rapp MA. Alcohol as an environmental mortality hazard. JAMA Psychiatry. 2016;73(6):549-550. doi:10.1001/jamapsychiatry.2016.0399 27096667

[3] World Health Organization. *Global Status Report on Alcohol and Health 2018.* World Health Organization; 2019. Accessed June 20, 2022. https://apps.who.int/iris/handle/10665/274603

[4] Naughton F, Ward E, Khondoker M, . Health behaviour change during the UK COVID-19 lockdown: findings from the first wave of the C-19 health behaviour and well-being daily tracker study. Br J Health Psychol. 2021;26(2):624-643. doi:10.1111/bjhp.12500 33410229 PMC9291054

[5] Pollard MS, Tucker JS, Green HD Jr. Changes in adult alcohol use and consequences during the COVID-19 pandemic in the US. JAMA Netw Open. 2020;3(9):e2022942. doi:10.1001/jamanetworkopen.2020.22942 32990735 PMC7525354

[6] Rubin R. Alcohol-related diseases increased as some people drank more during the COVID-19 pandemic. JAMA. 2021;326(3):209-211. doi:10.1001/jama.2021.10626 34190978

[7] Kim JU, Majid A, Judge R, . Effect of COVID-19 lockdown on alcohol consumption in patients with pre-existing alcohol use disorder. Lancet Gastroenterol Hepatol. 2020;5(10):886-887. doi:10.1016/S2468-1253(20)30251-X 32763197 PMC7403133

[8] Grossman ER, Benjamin-Neelon SE, Sonnenschein S. Alcohol consumption during the COVID-19 pandemic: a cross-sectional survey of US adults. Int J Environ Res Public Health. 2020;17(24):E9189. doi:10.3390/ijerph17249189 33316978 PMC7763183

[9] Yazdi K, Fuchs-Leitner I, Rosenleitner J, Gerstgrasser NW. Impact of the COVID-19 pandemic on patients with alcohol use disorder and associated risk factors for relapse. Front Psychiatry. 2020;11:620612. doi:10.3389/fpsyt.2020.620612 33391060 PMC7772314

[10] Trull TJ, Ebner-Priemer U. Ambulatory assessment. Annu Rev Clin Psychol. 2013;9:151-176. doi:10.1146/annurev-clinpsy-050212-185510 23157450 PMC4249763

[11] Trull TJ, Ebner-Priemer UW. Using experience sampling methods/ecological momentary assessment (ESM/EMA) in clinical assessment and clinical research: introduction to the special section. Psychol Assess. 2009;21(4):457-462. doi:10.1037/a0017653 19947780 PMC4255457

[12] Poulton A, Pan J, Bruns LR Jr, Sinnott RO, Hester R. Assessment of alcohol intake: retrospective measures versus a smartphone application. Addict Behav. 2018;83:35-41. doi:10.1016/j.addbeh.2017.11.003 29128148

[13] Dunton GF. Sustaining health-protective behaviors such as physical activity and healthy eating. JAMA. 2018;320(7):639-640. doi:10.1001/jama.2018.6621 29852046 PMC7524543

[14] Heinz A, Kiefer F, Smolka MN, ; Addiction Research Consortium. Addiction Research Consortium: losing and regaining control over drug intake (ReCoDe): from trajectories to mechanisms and interventions. Addict Biol. 2020;25(2):e12866. doi:10.1111/adb.12866 31859437

[15] Reichert M, Gan G, Renz M, . Ambulatory assessment for precision psychiatry: foundations, current developments and future avenues. Exp Neurol. 2021;345:113807. doi:10.1016/j.expneurol.2021.113807 34228998

[16] American Psychiatric Association. Diagnostic and Statistical Manual of Mental Disorders. 5th ed. 2013.

[17] Fydrich T, Sommer G, Brähler E. Fragebogen zur Sozialen Unterstützung: F-SozU; Manual. Hogrefe; 2007.

[18] Bundesregierung. Corona: Diese Regelungen gelten ab 2 November. Updated November 8, 2021. Accessed November 8, 2021. https://www.bundesregierung.de/breg-de/themen/coronavirus/regelungen-ab-2-november-1806818

[19] Bundesregierung. Lockdown: Diese Regeln gelten ab heute. Updated November 8, 2021. Accessed November 8, 2021. https://www.bundesregierung.de/breg-de/themen/coronavirus/bundesweiter-lockdown-1829134

[20] Bolger N, Laurenceau J-P. Intensive Longitudinal Methods: An Introduction to Diary and Experience Sampling Research. Guilford Press; 2013.

[21] Kuitunen-Paul S, Rehm J, Lachenmeier DW, . Assessment of alcoholic standard drinks using the Munich composite international diagnostic interview (M-CIDI): an evaluation and subsequent revision. Int J Methods Psychiatr Res. 2017;26(3):e1563. doi:10.1002/mpr.1563 28370786 PMC6877198

[22] Calina D, Hartung T, Mardare I, . COVID-19 pandemic and alcohol consumption: impacts and interconnections. Toxicol Rep. 2021;8:529-535. doi:10.1016/j.toxrep.2021.03.005 33723508 PMC7944101

[23] Tran TD, Hammarberg K, Kirkman M, Nguyen HTM, Fisher J. Alcohol use and mental health status during the first months of COVID-19 pandemic in Australia. J Affect Disord. 2020;277:810-813. doi:10.1016/j.jad.2020.09.012 33065821 PMC7476559

[24] Deeken F, Banaschewski T, Kluge U, Rapp MA. Risk and protective factors for alcohol use disorders across the lifespan. Curr Addict Rep. 2020;7(3):245-251. doi:10.1007/s40429-020-00313-z

[25] Imber S, Schultz E, Funderburk F, Allen R, Flamer R. The fate of the untreated alcoholic: toward a natural history of the disorder. J Nerv Ment Dis. 1976;162(4):238-247. doi:10.1097/00005053-197604000-00002 1255153

[26] Helzer JE, Robins LN, Taylor JR, . The extent of long-term moderate drinking among alcoholics discharged from medical and psychiatric treatment facilities. N Engl J Med. 1985;312(26):1678-1682. doi:10.1056/NEJM198506273122605 4000215

[27] Sobell LC, Ellingstad TP, Sobell MB. Natural recovery from alcohol and drug problems: methodological review of the research with suggestions for future directions. Addiction. 2000;95(5):749-764. doi:10.1046/j.1360-0443.2000.95574911.x 10885050

[28] Swift W, Coffey C, Degenhardt L, Carlin JB, Romaniuk H, Patton GC. Cannabis and progression to other substance use in young adults: findings from a 13-year prospective population-based study. J Epidemiol Community Health. 2012;66(7):e26. doi:10.1136/jech.2010.129056 21771817

[29] Mann K, Schäfer DR, Längle G, Ackermann K, Croissant B. The long-term course of alcoholism, 5, 10 and 16 years after treatment. Addiction. 2005;100(6):797-805. doi:10.1111/j.1360-0443.2005.01065.x 15918810

[30] Heron KE, Smyth JM. Ecological momentary interventions: incorporating mobile technology into psychosocial and health behaviour treatments. Br J Health Psychol. 2010;15(pt 1):1-39. doi:10.1348/135910709X466063 19646331 PMC2800172

