# Supplementary material for: Patterns of Alcohol Consumption Among Individuals With Alcohol Use Disorder During the COVID-19 Pandemic and Lockdowns in Germany
Source: JAMA Netw Open. 2022 Aug 1;5(8):e2224641. doi: 10.1001/jamanetworkopen.2022.24641 (PMC9344361; doi:10.1001/jamanetworkopen.2022.24641)
Supplement: Supplement 1. — eAppendix 1. Collaborative Research Center Grant 265 “ReCoDe” eAppendix 2. Participant Recruitment and Characteristics eAppendix 3. e-Diary Items eAppendix 4. Statistical Analyses and Results eAppendix 5. Main Multilevel Results eAppendix 6. Supportive Multilevel Analyses eReferences eTable 1. e-Diary List of Alcoholic Drinks eTable 2. Lockdown Measures in Germany eTable 3. Sex Distributed by Age and AUD Severity eTable 4. Main Multilevel Model Extended by Between-Person Predictors eTable 5. Holiday Season as an Additional Lockdown Phase Category eTable 6. Main Model With Log-Transformed Outcome eTable 7. Multilevel Results on Perceived Social Isolation eTable 8. Moderation Analyses Lockdown * Intention eTable 9. Moderation Analyses AUD Criteria * Weekend eTable 10. Moderation Analyses AUD Categories * Weekend eTable 11. Moderation Analyses Lockdown * Weekend eFigure 1. Repeated Real-life Assessment of (a) Perceived Social Isolation and (b) Drinking Intention eFigure 2. Study Flow Diagram eFigure 3. Development of Perceived Social Isolation (Y-Axis; Centered Within Subjects) During the Second Wave of the COVID-19 Pandemic in Germany eFigure 4. Level 1 Residuals [file jamanetwopen-e2224641-s001.pdf]

Supplementary Online Content

Deeken F, Reichert M, Zech H, et al; ReCoDe Consortium. Patterns of alcohol consumption among individuals with alcohol use disorder during the COVID-19 pandemic and lockdowns in Germany. *JAMA Netw Open*. 2022;5(8):e2224641. doi:10.1001/jamanetworkopen.2022.24641

eAppendix 1. Collaborative Research Center Grant 265 “ReCoDe”

eAppendix 2. Participant Recruitment and Characteristics

eAppendix 3. e-Diary Items

eAppendix 4. Statistical Analyses and Results

eAppendix 5. Main Multilevel Results

eAppendix 6. Supportive Multilevel Analyses

eReferences

eTable 1. e-Diary List of Alcoholic Drinks

eTable 2. Lockdown Measures in Germany

eTable 3. Sex Distributed by Age and AUD Severity

eTable 4. Main Multilevel Model Extended by Between-Person Predictors

eTable 5. Holiday Season as an Additional Lockdown Phase Category

eTable 6. Main Model With Log-Transformed Outcome

eTable 7. Multilevel Results on Perceived Social Isolation

eTable 8. Moderation Analyses Lockdown * Intention

eTable 9. Moderation Analyses AUD Criteria * Weekend

eTable 10. Moderation Analyses AUD Categories * Weekend

eTable 11. Moderation Analyses Lockdown * Weekend

eFigure 1. Repeated Real-life Assessment of (a) Perceived Social Isolation and (b) Drinking Intention

eFigure 2. Study Flow Diagram

eFigure 3. Development of Perceived Social Isolation (Y-Axis; Centered Within Subjects) During the Second Wave of the COVID-19 Pandemic in Germany

eFigure 4. Level 1 Residuals

This supplementary material has been provided by the authors to give readers additional information about their work.

© 2022 Deeken F et al. *JAMA Network Open.*

eAppendix 1. Collaborative Research Center Grant 265 "ReCoDe"

Data for this study were acquired within the Collaborative Research Center grant 265 "ReCoDe" (Losing and regaining control over drug intake), which started July 2019 in three centers in Germany (Charité Universitätsmedizin Berlin, Technical University Dresden, and Central Institute of Mental Health in Mannheim).[1] This research consortium has three main goals: (1) to identify triggers and modifying factors that longitudinally modulate the trajectories of losing and regaining control over drug consumption in real life, (2) to study underlying behavioral, cognitive, and neurobiological mechanisms, and (3) to implicate mechanism-based interventions. In the present study, data in the time before and within the "second lockdown" of the COVID-19 pandemic in Germany, i.e., from 10/02/2020 to 02/28/2021, was analyzed.

© 2022 Deeken F et al. *JAMA Network Open.*

eAppendix 2. Participant Recruitment and Characteristics

The recruitment process of this study had been continuous. The decision to start the analysis with the data beginning 10/02/20 was based on two considerations. First, for statistical power reasons and following multilevel analysis guidelines[4] as well as recent simulation studies[5], up to this point in time a sufficient number of participants (n = 105) had already been included in the study to be able to detect the hypothesized effects. Second, with the data beginning 10/02/20, the coverage of a sufficient time period across the second wave of the COVID19 pandemic in Germany was achieved, with roughly comparable length of time intervals across distinct lockdown stages: 4 weeks pre lockdown; 6 weeks light lockdown; 10 weeks hard lockdown.

We examined potential differences between included and excluded participants (see eFigure 2) according to age, gender, and AUD-criteria. In particular, we compared the sample of included participants with people eligible and with at least 2 AUD-criteria but wished for no further contact. To test differences in AUD criteria, we conducted a Mann-Whitney-U test and found no evidence that excluded participants fullfilled a higher number of AUD criteria compared to included participants (P =.419). Moreover, we found the excluded participants to be younger (median = 32 years (IQR, 24-43) than included participants (median = 37 years, IQR 27.5-52) (P=.003). Conducting a Chi^2-test, no significant difference was observed for the variable sex (P = .728).

None of the included participants dropped out during the 5 month study period. Data delivery differed between lockdown phases. In particular, 105 participants delivered data during the pre-lockdown, 146 participants during the light lockdown, and 189 participants during the hard lockdown. Multi-level models have been shown to be well suited to deal with data structure characterized by different amounts of data points.[4]

© 2022 Deeken F et al. *JAMA Network Open.*

eAppendix 3. e-Diary Items

The data frame used to compute the multilevel models was structured as follows. First, we restructured the main outcome variable of interest, i.e., alcohol intake rated every other day over the previous 2 days separately. This resulted in a continuous data set with a daily resolution, i.e., each row represented one day including the alcohol consumption at this particular day. Second, we restructured the intentions to drink item. In particular, this resulted in the time frame referenced (i.e., over 8 days) of the intention predictor preceding the time frame for the outcome (i.e., alcohol consumption). Third we restructured the social isolation item by extending it towards the preceding day.

© 2022 Deeken F et al. *JAMA Network Open.*

eAppendix 4. Statistical Analyses and Results

We estimated intraclass correlation coefficients (ICC) applying unconditional models. We received ICCs of 0.25 (AC), 0.60 (perceived social isolation), and 0.59 (drinking intention), indicating that about 75% of variance in AC, and each about 40% of variance in perceived social isolation and drinking intention was attributable to within-subject fluctuations (level 1 in our hierarchical statistical model).

We applied the same statistical procedure described for model 1 (see main manuscript for model 1 and equation 1), however, we entered perceived social isolation as being repeatedly reported by the participants on the e-diaries, as the dimensional outcome variable of interest as detailed in the following equation 2:

$$
\begin{aligned}
&Y(Perceived\ social\ isolation)_{ij} \\
&= \beta_{00} + \beta_{01} * age_j + \beta_{02} * gender_j + \beta_{03} * AUD\ criteria_j + \beta_{04} * study\ center_j + \beta_{10} \\
&\quad * weekend_{ij} + \beta_{20} * Christmas_{ij} + \beta_{30} * New\ Year's\ Eve_{ij} + \beta_{40} \\
&\quad * lockdown\ measures_{ij} + \beta_{50} * drinking\ intention_{ij} + \mu_{ij} + r_{ij}
\end{aligned}
$$

As depicted in eFigure 3, multilevel statistics confirmed a significant positive effect on perceived social isolation during the hard lockdown phase compared to pre lockdown (β-coefficient = 0.12; 95%CI, 0.06-0.15; $P < .001$; see eTable 7). In contrast, there was no significant difference between pre lockdown vs. light lockdown in perceived social isolation (β-coefficient = 0.06; 95%CI, 0.01-0.12; P =.019; eTable 7).

© 2022 Deeken F et al. *JAMA Network Open.*

eAppendix 5. Main Multilevel Results

Multilevel statistics showed that at weekends compared to weekdays, AC was significantly heightened (ß-coefficient = -11. 38; 95%CI, 10-12.77; *P* < .001; Table 2 and Figure 1 main text). Moreover, at Christmas as well as at New Year's Eve, significantly higher amounts of alcohol were consumed compared to all other days (ß-coefficient = 26.82; 95%CI, 21.87-31.77; *P* < .001; ß-coefficient = 66.88; 95%CI, 59.22-74-54, *P* < .001, respectively; Table 2 and Figure 1 main text). Perceived social isolation had no statistically significant effect on alcohol use (ß-coefficient = -1.31; 95%CI, -2.89-0.27; *P* = .104). During the lockdown phase with hard restrictions, AC was decreased by 5.45 gram compared to pre lockdown (ß-coefficient = -5.45; 95%CI, -8 – -2.9, *P* = .001; Table 2 and Figure 1 main text). Between pre lockdown and the light lockdown, no significant difference on AC was found (ß-coefficient = -1.30; 95%CI, -3.94-1.33; *P* = .333; Table 2 and Figure 1 main text). To assess the difference between the light lockdown and the hard lockdown phase, the same model was performed with the light lockdown as the reference group resulting in a significantly lower AC during the hard lockdown compared to the light lockdown (ß-coefficient = -4.15; 95%CI, -5.95 - -2.35, *P* < .001). Drinking intention was negatively associated with AC (ß-coefficient (no more AC than usual) = -3.97; 95%CI, -6.56 - -1.38; *P* = 0.003; ß-coefficient (less AC than usual) = -11.10; 95%CI, -13.63 - -8.58; *P* < .001; reference = no particular resolutions; Table 2 main text), indicating that when participants intended to consume less alcohol, this resulted in a reduction in the amount of alcohol consumed. Translated to practice, if participants plans to limit their "alcohol consumption for the next eight days?" were indicated as „Yes, I want to drink not more than usual" or „Yes, I want to drink less than usual", these ratings were associated with less alcohol consumption self-reported every other day compared to participants response „No, I don´t have any particular resolutions".

Moderation analyses

To test whether the effect of drinking intention was different across lockdown measures, we applied multilevel moderation analysis, see equation 3:

$$Y(Alcohol\ consumption)_{ij} = \beta_{00} + \beta_{10} * lockdown\ measures_{ij} + \beta_{20} * drinking\ intention_{ij} + \beta_{30} * lockdown\ measures_{ij} * drinking\ intention_{ij} + \mu_{ij} + r_{ij}$$

These analyses of the interaction effect of intention to drink alcohol and lockdown measures on AC revealed no significant effect (*F*(3967) = 0.63; *P* = .638), neither for intention and light lockdown, nor for intention and the hard lockdown (eTable 8). The results indicate that the intention to drink alcohol had a significant effect on consumption regardless of the lockdown measures. Please note that, following established procedures to maximize statistical power in multilevel moderation analyses,[4,5] we computed interactions in models with a reduced set of predictors.

© 2022 Deeken F et al. *JAMA Network Open.*

To explore whether weekend drinking cycles of AC were influenced by AUD severity, we computed a multilevel moderation analysis as detailed in the following equation 4:

$$Y(Alcohol\ consumption)_{ij} = \beta_{00} + \beta_{01} * AUD\ criteria_{j} + \beta_{10} * weekend_{ij} + \beta_{20} * AUD\ criteria_{j} * weekend_{ij} + \mu_{ij} + r_{ij}$$

We found a significant interaction effect for AUD criteria * weekend ($F(12e^3) = 5.02$; $P = .025$), i.e., participants with more AUD criteria showed a smaller difference of AC between weekend days and weekdays (eTable 9).

For interpretation and visualization purposes (see Figure 2a in the main text), we recomputed the moderation analyses AUD criteria * weekend using AUD categories of the DSM-5[7] (2-3 criteria = mild, 4-5 criteria = moderate, ≥ 6 criteria = severe). Again, we found a significant interaction effect ($F(12e^3) = 4.89$; $P = .008$) revealing significant differences between AC at weekend days vs. weekdays in the mild (difference in AC: 12.86 grams alcohol per day; SE = 0.93; $P < .001$), moderate (difference in AC: 11.50 grams alcohol per day; SE = 1.03; $P < .001$) and severe AUD groups (difference in AC: 6.60 grams alcohol per day; SE = 1.77; $P < .001$), respectively. The interaction effect was driven by differences between the mild and severe AUD groups (eTable 10).

To enable a more fine-grained interpretation we plotted the simple effects, i.e., AC at weekdays and weekend days by AUD category (Figure 3a). To explore whether weekend drinking cycles of AC were influenced by lockdown measures, we computed a further multilevel moderation analysis as detailed in the following equation 5:

To enable a more fine-grained interpretation we plotted the simple effects, i.e., AC at weekdays and weekend days by lockdown phase (Figure 3b in the main manuscript).

We conducted three supplementary interaction analyses to examine a potential moderating role of age on the associations between i) lockdown measures, ii) weekend drinking cycles, iii) intention to drink and AC. While there was no moderation effect of age on the associations intention to drink and AC ($F(4161) = 0.03$; $P = .9732$), age significantly moderated the associations between lockdown measures and AC ($F(4337) = 3.68$; $P = .0252$); i.e., the older the participants were, the smaller the reduction of AC in the lockdown phases was. Moreover, age significantly moderated the association between weekend drinking cycles and AC with reduced weekday vs weekend AC differences as a function of increasing age ($F(12000) = 12.72$; $P < .001$); i.e., the older the participants, the smaller the weekday vs weekend AC difference was.

Holiday season as an additional lockdown phase

We conducted a supplementary analysis treating the hard lockdown as consisting of two distinct time periods. In particular, we extended our predictor lockdown and introduced the time periods between the build-up to Christmas and New Years ((2020/16/12) to (2021/01/02)) as an additional category. Results showed no difference between pre lockdown and the “holiday phase”, but again confirmed the reduced consumption during the hard lockdown (see eTable 5)

Multi-level model with log-transformed AC outcome

Given the daily life consumption behavior of our sample (i.e., the at risk yet not highly alcohol-dependent participants did not consume alcohol each and every day), AC as the main outcome variable of interest is skewed and does show outliers. Therefore, we conducted a robustness check. Following established procedures for transforming such skewed data,[4,8,9] we log-transformed all AC-values using the natural logarithm and adding “1”: $\log_n$ (AC-values + 1). We recomputed the multi-level model and received the contentual same results compared

© 2022 Deeken F et al. *JAMA Network Open.*

to the multilevel model with non-transformed AC- values (see eTable 6). To judge whether this multilevel model is suited for dealing with the given data structure, we followed established procedures[4] and thus examined level-1 (assessment-level) residuals measuring deviations from the conditional mean (conditional residuals) which we derived from our multilevel model. Visual inspection confirmed that there was no obvious deviation from a normal distribution of the residuals. (see eFigure 4).

© 2022 Deeken F et al. *JAMA Network Open.*

eAppendix 6. Supportive Multilevel Analyses

Controlling for between person effects

Following Wang and Maxwell (2015)[6], we computed a multilevel model including all between-person effects (i.e., each within-person predictor aggregated on a between-person mean value, respectively) as a supplementary analysis. None of the between-person predictors yielded significant effects (see eTable 4).

© 2022 Deeken F et al. *JAMA Network Open.*

eReferences

1. Heinz A, Kiefer F, Smolka MN, et al. Addiction Research Consortium: Losing and regaining control over drug intake (ReCoDe)—From trajectories to mechanisms and interventions. *Addiction Biology*. 2020;25(2):e12866. doi:10.1111/adb.12866.

2. Bundesregierung. Corona: Diese Regelungen gelten ab 2. November. https://www.bundesregierung.de/breg-de/themen/coronavirus/regelungen-ab-2-november-1806818. Updated November 8, 2021.000Z. Accessed November 8, 2021.542Z.

3. Bundesregierung. Lockdown: Diese Regeln gelten ab heute. https://www.bundesregierung.de/breg-de/themen/coronavirus/bundesweiter-lockdown- 1829134. Updated November 8, 2021.000Z. Accessed November 8, 2021.535Z.

4. Bolger N, Laurenceau J-P. *Intensive Longitudinal Methods: An Introduction to Diary and Experience Sampling Research*. Guilford Press; 2013.

5. Arend MG, Schäfer T. Statistical power in two-level models: A tutorial based on Monte Carlo simulation. *Psychological Methods*. 2019;24(1):1-19. doi:10.1037/met0000195.

6. Wang LP, Maxwell SE. On disaggregating between-person and within-person effects with longitudinal data using multilevel models. *Psychol Methods*. 2015;20(1):63-83. 249 doi:10.1037/met0000030.

7. American Psychiatric Association. *Diagnostic and Statistical Manual of Mental Disorders (5th ed.)*. Washington, DC; 2013.

8. Santangelo P, Reinhard I, Mussgay L, et al. Specificity of affective instability in patients with borderline personality disorder compared to posttraumatic stress disorder, bulimia nervosa, and 254 healthy controls. *J Abnorm Psychol*. 2014;123(1):258-272. doi:10.1037/a0035619.

9. Reichert M, Tost H, Reinhard I, et al. Within-Subject Associations between Mood Dimensions and Non-exercise Activity: An Ambulatory Assessment Approach Using Repeated Real-Time and Objective Data. *Front Psychol*. 2016;7:918. doi:10.3389/fpsyg.2016.00918.

© 2022 Deeken F et al. *JAMA Network Open.*

eTable 1. e-Diary List of Alcoholic Drinks

no alcoholic drink
small beer<br>0,2l / 5,0% ABV*
regular beer<br>0,33l / 5,0% ABV
large beer<br>0,5l / 5,0% ABV
small white wine<br>0,1l / 11,0% ABV
regular white wine<br>0,2l / 11,0% ABV
bottle of white wine<br>0,75l / 11,0% ABV
small red wine<br>0,1l / 12,0% ABV
regular red wine<br>0,2l / 12,0% ABV
bottle of red wine<br>0,75l / 12,0% ABV
sparkling wine<br>0,1l / 11,5% ABV
bottle of sparkling wine<br>0,75l / 11,5% ABV
fortified wine (e.g. port, Sherry)<br>5cl / 19,7% ABV
small liqueur (e.g. Cointreau, Sambuca, Underberg, Jägermeister)<br>2cl / 20% ABV
large liqueur (e.g. Cointreau, Sambuca, Underberg, Jägermeister)<br>5cl / 20% ABV
sweet liqueur (e.g. Aperol, Amaretto, Advocaat, Baileys, Batida de Coco)<br>2cl / 17% ABV
small spirit (e.g. fruit liqueur, cherry liqueur, Vodka, Gin,<br>juniper, corn brandy) 2cl / 40% ABV
large spirit (e.g. fruit liqueur, kirsch, Vodka, Gin, juniper, corn brandy)<br>5cl / 40% ABV
spirit (e.g. fruit liqueur, kirsch, Vodka, Gin, juniper, corn brandy)<br>100ml / 40% ABV
small spirit (strong)<br>2cl / 65% ABV
Brandy (e.g. Brandy, Cognac, Armagnac, Metaxa)<br>2cl / 36% ABV

*ABV = Alcohol by volume

© 2022 Deeken F et al. *JAMA Network Open.*

eTable 2. Lockdown Measures in Germany

pre lockdown (10/02/2020-11/01/2020)	Adherence to the **AHA** guideline [**A**bstand, **H**ygiene und **A**lltagsmaske translated: distance, hygiene, and mask] is promoted
light lockdown (11/02/2020-12/15/2020)	- In principle, staying in public is allowed only with the members of the own and one other household, a **maximum of 10 people** may come together.<br>- Citizens are asked to generally refrain from non-essential private travel and visits, including those of relatives. Overnight accommodations within the country will be made available only for necessary travel and explicitly non-touristic purposes.<br>- Institutions and facilities that are classified as recreational are to be closed, including recreational and amateur sporting operations at and in all public and private sports facilities - with the exception of individual sports alone, in pairs or with members one's own household only.<br>- Restaurants and bars, clubs, pubs and similar establishments are to be closed. This excludes the delivery and collection of takeaway food for consumption at home and the operating canteens in workplaces. [2]
hard lockdown (12/16/2020-02/28/2021)	- Private meetings are limited to a **maximum of 5 people** from a maximum of two households for private meetings.<br>- Retail and service businesses, such as hair salons and beauty salons, are to be closed from December 16. Exceptions apply to grocery stores, drugstores, pharmacies, opticians, gas stations, auto repair shops, banks, post offices, dry cleaners and Christmas tree dealers. Medically necessary treatments such as physical therapy also remain available.<br>- At schools, contacts are to be significantly restricted from Dec. 16 until Jan. 10, 2021. Children are to be cared for at home whenever possible during this period. Therefore, schools will generally be closed or attendance will be suspended during this period. Emergency care is provided and distance learning is offered. An analogous approach is taken in daycare centers. Additional opportunities are created for parents to take paid leave for childcare.<br>- Employers are urged to consider whether business sites can be closed either through company vacations or generous home office solutions from Dec. 16 to Jan. 10, 2021.<br>- In the food service industry, delivery and pickup of take-out meals for home consumption by food service establishments will remain available. On-site consumption is prohibited. **Consumption of alcoholic beverages in public areas is prohibited** from Dec. 16 to Jan. 10. Violators are to be fined. [3]

© 2022 Deeken F et al. *JAMA Network Open.*

eTable 3. Sex Distributed by Age and AUD Severity

**Characteristics**	**Men**	**Women**
Age		
16-32	47	27
33-49	37	21
50-65	35	22
AUD Criteria		
2 criteria	30	10
3 criteria	27	23
4 criteria	22	11
5 criteria	25	16
6 criteria	11	7
7 criteria	4	3

© 2022 Deeken F et al. *JAMA Network Open.*

eTable 4. Main Multilevel Model Extended by Between-Person Predictors

Outcome: Alcohol consumption

Predictor	β coefficient (SE; 95% CI)	t(df)	P
Intercept	46.51 (81.77; -114.11-207.14)	0.57 (533)	.570
Age	0.44 (0.15; 0.15 – 0.72)	3.00 (178)	.003
Sex			
Male	0 [Reference]	0 [Reference]	0 [Reference]
Female	-9.28 (3.58; -16.35 - -2.22)	-2.59 (176)	.010
AUD criteria	2.63 (1.31; 0.056 – 5.21)	2.02 (175)	.045
Study center			
CI of Mental Health in Mannheim	0 [Reference]	0 [Reference]	0 [Reference]
Charité Berlin	-2.94 (3.95; -10.74 – 4.87))	-0.74 (173)	.459
TU Dresden	1.45 (5.46; -9.33 – 12.23)	0.27 (178)	.791
Weekend vs weekday			
Weekday	0 [Reference]	0 [Reference]	0 [Reference]
Weekend	11.40 (0.71; 10.01 – 12.79)	16.11 (9013)	< .001
Weekend vs weekday person mean	-128.42 (191.41; -504.43 – 247.58)	-0.67 (540)	.503
Christmas			
No Christmas, i.e. all other days	0 [Reference]	0 [Reference]	0 [Reference]
Christmas	26.74 (2.53; 21.79 – 31.69)	10.59 (6130)	< .001
Christmas person mean	602.62 (436.69; -258.93 – 1479.89)	1.38 ($11e^3$)	.169
New Year´s Eve			
No New Year´s Eve, i.e. all other days	0 [Reference]	0 [Reference]	0 [Reference]
New Year´s Eve	66.79 (3.91; 59.12 – 74.45)	17.08 ($11e^3$)	< .001
New Year´s Eve person mean	-869.82 (11191; -3219.53 – 1479.89)	-0.73(185)	.466
Lockdown stage			
Pre lockdown	0 [Reference]	0 [Reference]	0 [Reference]
Light lockdown	-1.35 (1.35; -3.99 – 1.29)	-1.00 (3528)	.316
Hard lockdown	-5.48 (1.31; -8.04 - -2.92)	-4.19 (3476)	<.001
Lockdown person mean	6.89 (7.63; -8.15 – 21.94)	0.90 (213)	.367
Perceived social isolation	-1.32 (0.81; -2.90 – 0.26)	-1.64 (6862)	.102
Perceived social isolation person mean	3.02 (3.18; -3.25 – 9.3)	0.95(185)	0.343
Intention			
No particular resolutions	0 [Reference]	0 [Reference]	0 [Reference]
No more AC than usual	-4.07 (1.34; -6.69 - -1.45)	-3.04 (3910)	.002
Less AC than usual	-11.16 (1.32; -13.75 - -8.57)	-8.44 (3824)	< .001
Intention person mean	1.95 (2.97; -3.9 – 7.8)	0.66 (216)	.511

© 2022 Deeken F et al. *JAMA Network Open.*

eTable 5. Holiday Season as an Additional Lockdown Phase Category
Outcome: Alcohol consumption

Predictor	β coefficient (SE; 95% CI)	t(df)	P
Intercept	10.07 (7.80; -5.31 – 25.45)	1.29 (188)	.198
Age	0.47 (0.14; 0.20 – 0.74)	3.39 (183)	< .001
Sex			
Male	0 [Reference]	0 [Reference]	0 [Reference]
Female	-9.68 (3.51; -16.59 - -2.76)	-2.76 (181)	.006
AUD criteria	3.39 (1.17; 1.08 – 5.71)	2.90 (179)	.004
Study center			
CI of Mental Health in Mannheim	0 [Reference]	0 [Reference]	0 [Reference]
Charité Berlin	-3.31 (3.88; -10.97 – 4.36)	-0.85 (178)	.396
TU Dresden	0.10 (5.35; -10.46 – 10.67)	0.02 (183)	.985
Weekend vs weekday			
Weekday	0 [Reference]	0 [Reference]	0 [Reference]
Weekend	11.37 (0.70; 9.99 – 12.75)	16.13 (8968)	< .001
Christmas			
No Christmas, i.e. all other days	0 [Reference]	0 [Reference]	0 [Reference]
Christmas	20.86 (2.67; 15.62 – 26.10)	7.80 (69499	< .001
New Year´s Eve			
No New Year´s Eve, i.e. all other days	0 [Reference]	0 [Reference]	0 [Reference]
New Year´s Eve	61.07 (4.00; 53.22 – 68.91)	15.26 ($11e^3$)	< .001
Lockdown stage			
Pre lockdown	0 [Reference]	0 [Reference]	0 [Reference]
Light lockdown	-1.01 (1.34; -3.64 – 1-61)	-0.76 (3551)	.450
Hard lockdown	-7.29 (1.32; -9.87 - -4.70)	-5.53 (3502)	<.001
Holiday phase	1.30 (1.66; -1.95 – 4.55)	0.78 (4069)	.433
Perceived social isolation	-1.21 (0.80; -2.79 – 0.36)	-1.51 (6835)	.130
Intention			
No particular resolutions	0 [Reference]	0 [Reference]	0 [Reference]
No more AC than usual	-3.37 (1.32; -5.95 - -0.78)	-2.55 (3948)	.012
Less AC than usual	-9.99 (1.29; -12.53 - -7.46)	-7.74 (3987)	< .001

© 2022 Deeken F et al. *JAMA Network Open.*

eTable 6. Main Model With Log-Transformed Outcome

Outcome: Alcohol consumption log transformed

Predictor	β coefficient (SE; 95% CI)	t(df)	P
Intercept	0.50 (0.35; -0.19 – 1.20)	1.42 (181)	.156
Age	0.04 (0.01; 0.03 – 0.05)	6.34 (176)	< .001
Sex			
Male	0 [Reference]	0 [Reference]	0 [Reference]
Female	-0.26 (0.16; -0.57 – 0.05)	-1.63 (174)	.104
AUD criteria	0.12 (0.05; 0.02 – 0.23)	2.29 (172)	.023
Study center			
CI of Mental Health in Mannheim	0 [Reference]	0 [Reference]	0 [Reference]
Charité Berlin	-0.07 (0.18; -0.41 – 0.28)	-0.83 (171)	.701
TU Dresden	0.14 (0.24; -0.33 – 0.62)	0.59 (176)	.554
Weekend vs weekday			
Weekday	0 [Reference]	0 [Reference]	0 [Reference]
Weekend	0.53 (0.03; 0.47 – 0.60)	16.50 (9267)	< .001
Christmas			
No Christmas, i.e. all other days	0 [Reference]	0 [Reference]	0 [Reference]
Christmas	0.92 (0.12; 0.69 – 1.15)	7.89 (6296)	< .001
New Year´s Eve			
No New Year´s Eve, i.e. all other days	0 [Reference]	0 [Reference]	0 [Reference]
New Year´s Eve	1.90 (0.18; 1.56 – 2.259	10.72 ($11e^3$)	< .001
Lockdown stage			
Pre lockdown	0 [Reference]	0 [Reference]	0 [Reference]
Light lockdown	-0.10 (0.06; -0.22 – 0.02)	-1.58 (3490)	.114
Hard lockdown	-0.29 (0.06; -0.41 - -0.17)	-4.75 (34729	<.001
Perceived social isolation	-0.07 (0.04; -0.14 – 0.01)	-1.79 (70559	.074
Intention			
No particular resolutions	0 [Reference]	0 [Reference]	0 [Reference]
No more AC than usual	-0.16 (0.06; -0.28 - -0.04)	-2.58 (3916)	.010
Less AC than usual	-0.57 (0.06; -0.69 - -0.45)	-9.51 (3950)	< .001

© 2022 Deeken F et al. *JAMA Network Open.*

eTable 7. Multilevel Results on Perceived Social Isolation

Outcome: Perceived social isolation			
Predictor	β coefficient (SE; 95% CI)	t(df)	P
Intercept	0.91 (0.18, 0.55-1.27)	5.02 (186)	<.001
Age	0.00 (0.00, -0.01-0.01)	0.31 (182)	.757
Sex			
Male	0 [Reference]	0 [Reference]	0 [Reference]
Female	0.10 (0.08, -0.06-0.26)	1.20 (185)	.233
AUD criteria	0.10 (0.03, 0.04-0.15)	3.31 (180)	.001
Study center			
CI of Mental Health in Mannheim	0 [Reference]	0 [Reference]	0 [Reference]
Charité Berlin	0.01 (0.09, -0.17-0.19)	0.15 (182)	.193
TU Dresden	-0.08 (0.13, -0.33-0.17)	-0.65 (183)	.517
Weekend vs weekday			
Weekday	0 [Reference]	0 [Reference]	0 [Reference]
Weekend	-0.03 (0.01, -0.04- -0.01)	-4.07 ($11e^3$)	<.001
Christmas			
No Christmas, i.e. all other days	0 [Reference]	0 [Reference]	0 [Reference]
Christmas	-0.09 (0.03, -0.15- -0.03)	-3.01 ($12e^3$)	.003
New Year´s Eve			
No New Year´s Eve, i.e. all other days	0 [Reference]	0 [Reference]	0 [Reference]
New Year´s Eve	-0.03 (0.04, -0.12-0.04)	-0.87 ($12e^3$)	.386
Lockdown stage			
Pre lockdown	0 [Reference]	0 [Reference]	0 [Reference]
Light lockdown	0.06 (0.03, 0.01-0.12)	2.34 (2766)	.019
ard lockdown	0.12 (0.03, 0.06-0.15)	4.20 (2394)	<.001
Intention			
No particular resolutions	0 [Reference]	0 [Reference]	0 [Reference]
No more AC than usual	-0.01 (0.02, -0.05-0.03)	-0.40 (5460)	.693
Less AC than usual	-0.00 (0.02, -0.04-0.04)	-0.04 (5258)	.972

© 2022 Deeken F et al. *JAMA Network Open.*

eTable 8. Moderation Analyses Lockdown * Intention

Outcome: Alcohol consumption

Predictor	β coefficient (SE; 95% CI)	t(df)	P
Intercept	44.23 (2.46; 39.42-49.05)	18.01 (631)	<.001
Lockdown stage			
Pre lockdown	0 [Reference]	0 [Reference]	0 [Reference]
Light lockdown	-4.51 (1.96; -8.35- -0.67=	-2.30 (3785)	.021
Hard lockdown	-4.45 (1.89; -8.15- -0.75)	-2.36 (3738)	.018
Intention			
No particular resolutions	0 [Reference]	0 [Reference]	0 [Reference]
No more AC than usual	-7.36 (3.09; -13.42- -1.29)	-2.38 (3998)	.017
Less AC than usual	-13.33 (2.89; -19.02- -7.65)	-4.60 (4130)	<.0001
Pre lockdown * Intention	0 [Reference]	0 [Reference]	0 [Reference]
Light lockdown* Intention: no more AC than usual	3.18 (3.42; -3.52-9.90)	0.93 (3950)	.352
Light lockdown* Intention: less AC than usual	2.58 (3.34; -3.96-9.13)	0.77 (4111)	.440
Hard lockdown * Intention: no more AC than usual	2.54 (3.28; -3.89-8.96)	0.77 (3917)	.439
Hard lockdown * Intention: less AC than usual	-0.43 (3.13; -6.56-5.70)	-0.14 (4068)	.890

© 2022 Deeken F et al. *JAMA Network Open.*

eTable 9. Moderation Analyses AUD Criteria * Weekend

Outcome: Alcohol consumption

Predictor	β coefficient (SE; 95% CI)	t(df)	P
Intercept	19.45 (4.75; 10.09-28.81)	4.10 (200)	<.001
AUD criteria	2.66 (1.16; 0.37-4.94)	2.29 (200)	.023
Weekend vs weekday			
Weekday	0 [Reference]	0 [Reference]	0 [Reference]
Weekend	15.24 (1.79; 11.73-18.74)	8.52 ($12e^3$)	<.001
AUD criteria * Weekday	0 [Reference]	0 [Reference]	0 [Reference]
AUD criteria * Weekend	-0.99 (0.44; -1.85- -0.12)	-2.24 ($12e^3$)	.025

© 2022 Deeken F et al. *JAMA Network Open.*

eTable 10. Moderation Analyses AUD Categories * Weekend

Outcome: Alcohol consumption

Predictor	β coefficient (SE; 95% CI)	t(df)	P
Intercept	26.62 (2.43; 21.83-31.40)	10.97 (204)	<.001
AUD			
Mild	0 [Reference]	0 [Reference]	0 [Reference]
Moderate	5.22 (3.60; -1.88-12.31)	1.45 (203)	.149
Severe	7.03 (5.17; -3.17-17.22)	1.36 (201)	.176
Weekend vs weekday			
Weekday	0 [Reference]	0 [Reference]	0 [Reference]
Weekend	12.86 (0.93; 11.03-14.68)	13.81 ($12e^3$)	<.001
AUD criteria * Weekday	0 [Reference]	0 [Reference]	0 [Reference]
Moderate AUD * Weekend	-1.36 (1.39; -4.09-1.37)	-0.98 ($12e^3$)	.329
Severe AUD * Weekend	-6.26 (2.00; -10.18- -2.34)	-3.13 ($12e^3$)	.002

© 2022 Deeken F et al. *JAMA Network Open.*

eTable 11. Moderation Analyses Lockdown * Weekend

Outcome: Alcohol consumption

Predictor	β coefficient (SE; 95% CI)	t(df)	P
Intercept	30.69 (2.08; 26.61-34.77)	14.78 (488)	<.001
Weekend vs weekday			
Weekday	0 [Reference]	0 [Reference]	0 [Reference]
Weekend	15.04 (1.76; 11.58-18.49)	8.53 ($12e^3$)	<.0001
Lockdown stage			
Pre lockdown	0 [Reference]	0 [Reference]	0 [Reference]
Light lockdown	-0.92 (1.54; -3.93-2.11)	-0.59 (6162)	.556
Hard lockdown	-1.12 (1.47; -4.00-1.77)	-0.76 (6059)	.447
Pre Lockdown * Weekend	0 [Reference]	0 [Reference]	0 [Reference]
Light Lockdown * Weekend	0.36 (2.15; -3.85-4.56)	0.17 ($12e^3$)	.868
Hard Lockdown * Weekend	-6.12 (1.95; -9.96- -2.31)	-3.14 ($12e^3$)	.002

© 2022 Deeken F et al. *JAMA Network Open.*

a

b

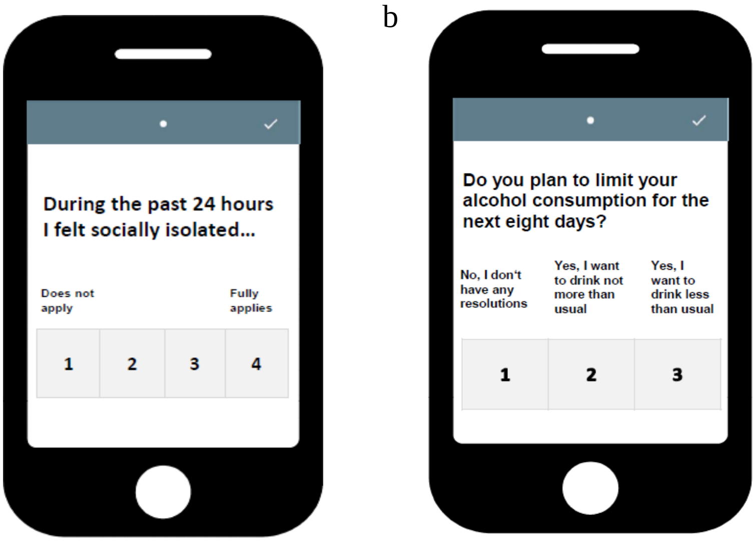

eFigure 1. Repeated Real-life Assessment of (a) Perceived Social Isolation and (b) Drinking Intention

© 2022 Deeken F et al. *JAMA Network Open.*

eFigure 2. Study Flow Diagram

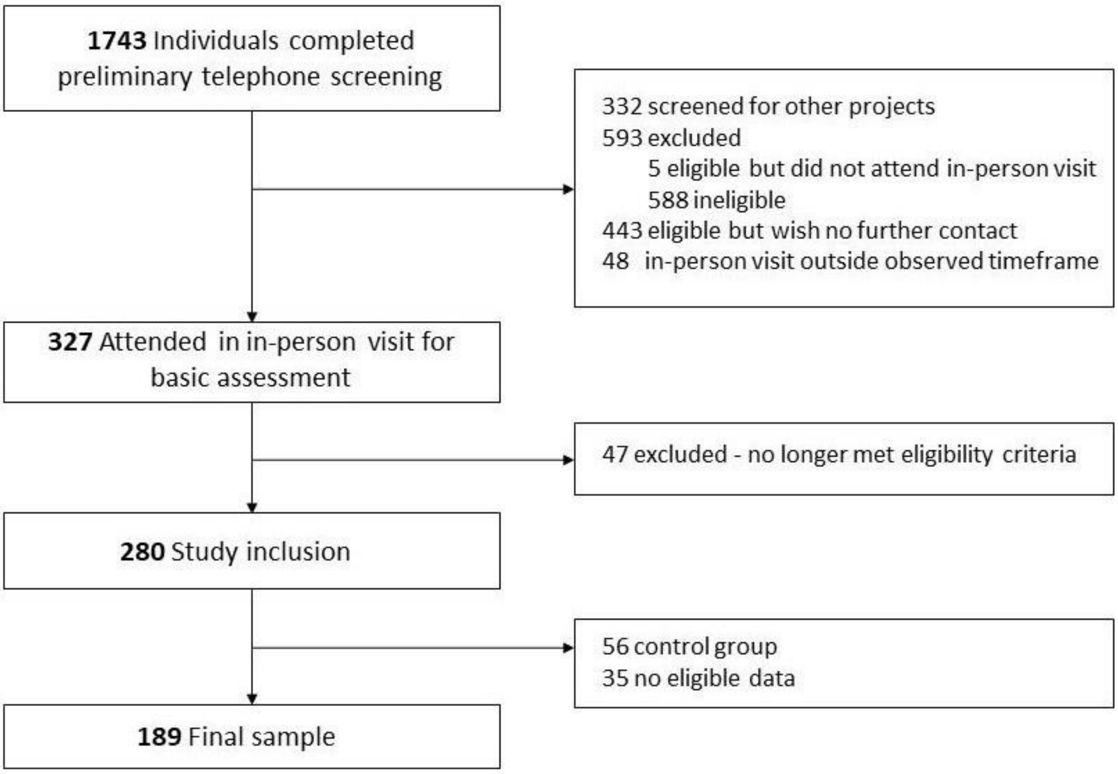

© 2022 Deeken F et al. *JAMA Network Open.*

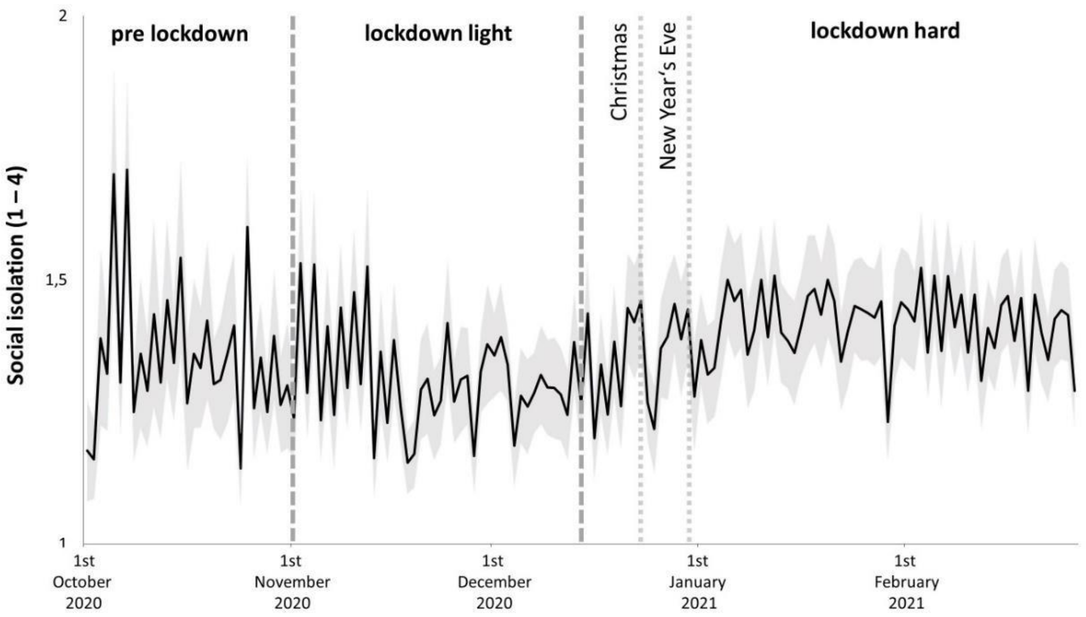

eFigure 3. Development of Perceived Social Isolation (Y-Axis; Centered Within Subjects) During the Second Wave of the COVID-19 Pandemic in Germany

Lockdown measures (beginning of the light lockdown and beginning of the hard lockdown) and holidays (Christmas and New Year's Eve) are highlighted (see x-axis).

© 2022 Deeken F et al. *JAMA Network Open.*

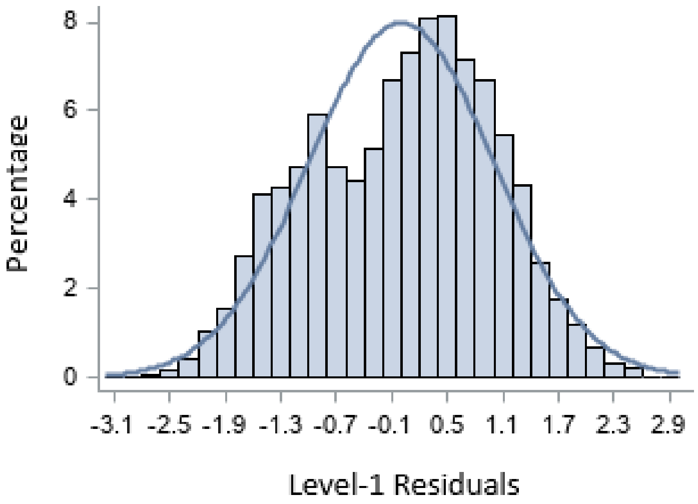

eFigure 4. Level 1 Residuals

Assessment-level residuals measuring deviations from the conditionalmean (conditional residuals).

© 2022 Deeken F et al. *JAMA Network Open.*
