# Supplementary material for: Patterns of Alcohol Consumption Among Individuals With Alcohol Use Disorder During the COVID-19 Pandemic and Lockdowns in Germany
Source: JAMA Netw Open. 2022 Aug 1;5(8):e2224641. doi: 10.1001/jamanetworkopen.2022.24641 (PMC9344361; doi:10.1001/jamanetworkopen.2022.24641)
Supplement: Supplement 2. — Nonauthor Collaborators [file jamanetwopen-e2224641-s002.pdf]

Supplemental Online Content: Nonauthor Collaborators
*First name, last name, and suffix (if applicable) are required and will appear in PubMed.

*Group Name(s): ReCoDe Consortium

*First Name and Middle Initial(s)	*Last Name	*Suffix (eg, Jr, III)	Academic Degrees	Institution	Location (city, state/province, country)	Role or Contribution, eg, chair, principal investigator	Group (if more than 1 Group listed in the byline) and/or Subgroup (eg, Steering Committee)
Nina	Romanczuk-Seiferth		Dr. rer. medic.	Charité Berlin	Berlin, Germany	Project-PI	ReCoDe-Consortium
Andreas	Ströhle		MD	Charité Berlin	Berlin, Germany	Project-PI	ReCoDe-Consortium
Felix	Bermpohl		MD	Charité Berlin	Berlin, Germany	Project-PI	ReCoDe-Consortium
Christine	Heim		Dr. rer. nat.	Charité Berlin	Berlin, Germany	Project-PI	ReCoDe-Consortium
Anne	Beck		Dr. rer. medic.	Health and Medical University Potsdam	Potsdam, Germany	Project-PI	ReCoDe-Consortium
Heike	Tost		MD, PhD	ZI Mannheim	Mannheim, Germany	Project-PI	ReCoDe-Consortium
Tobias	Banaschewski		MD, Dr. rer. nat.	ZI Mannheim	Mannheim, Germany	Project-PI	ReCoDe-Consortium
Falk	Kiefer		MD	ZI Mannheim	Mannheim, Germany	Project-PI	ReCoDe-Consortium
Andreas	Meyer-Lindenberg		MD, PhD	ZI Mannheim	Mannheim, Germany	Project-PI	ReCoDe-Consortium
Christian	Beste		Dr. rer. nat.	TU Dresden	Dresden, Germany	Project-PI	ReCoDe-Consortium
Tanja	Endraß		Dr. rer. nat.	TU Dresden	Dresden, Germany	Project-PI	ReCoDe-Consortium
Stefan	Kiebel		Dr. rer. nat.	TU Dresden	Dresden, Germany	Project-PI	ReCoDe-Consortium
Clemens	Kirschbaum		Dr. rer. nat.	TU Dresden	Dresden, Germany	Project-PI	ReCoDe-Consortium
Michael	Marxen		PhD	TU Dresden	Dresden, Germany	Project-PI	ReCoDe-Consortium
Wolfgang E.	Nagel		Dr. rer. nat.	TU Dresden	Dresden, Germany	Project-PI	ReCoDe-Consortium
Maximilian	Pilhatsch		MD	TU Dresden	Dresden, Germany	Project-PI	ReCoDe-Consortium
Ann-Kathrin	Stock		Dr. rer. nat.	TU Dresden	Dresden, Germany	Project-PI	ReCoDe-Consortium
Viktoria	Arndt		Dipl.-Psych.	TU Dresden	Dresden, Germany	Co-Investigator	ReCoDe-Consortium
Matthew	Belanger		M.Sc.	TU Dresden	Dresden, Germany	Co-Investigator	ReCoDe-Consortium
Hao	Chen		M.Sc.	TU Dresden	Dresden, Germany	Co-Investigator	ReCoDe-Consortium
Sasha	Frölich		M.Sc.	TU Dresden	Dresden, Germany	Co-Investigator	ReCoDe-Consortium
Filippo	Ghin		M.Sc.	TU Dresden	Dresden, Germany	Co-Investigator	ReCoDe-Consortium
Caroline	Neumer		M.Sc.	TU Dresden	Dresden, Germany	Co-Investigator	ReCoDe-Consortium
Sarah	Schwöbel		Dr. rer. nat.	TU Dresden	Dresden, Germany	Co-Investigator	ReCoDe-Consortium
Gabriela	Gan		PhD	ZI Mannheim	Mannheim, Germany	Co-Investigator	ReCoDe-Consortium
Kristina	Schwarz		Dipl.-Psych.	ZI Mannheim	Mannheim, Germany	Co-Investigator	ReCoDe-Consortium
Stefanie	Kunas		M.Sc.	Charité Berlin	Berlin, Germany	Co-Investigator	ReCoDe-Consortium
Heiner	Stuke		MD	Charité Berlin	Berlin, Germany	Co-Investigator	ReCoDe-Consortium
